# Supplementary material for: Practice of hyperglycaemia control in intensive care units of the Military Hospital, Sudan—Needs of a protocol
Source: PLoS One. 2022 May 24;17(5):e0267655. doi: 10.1371/journal.pone.0267655 (PMC9129021; doi:10.1371/journal.pone.0267655)
Supplement: S1 Table — (DOCX) [file pone.0267655.s001.docx]

**Table S1: Awareness of health care staff towards hyperglycaemia control methods and the reasons for lack of awareness (n=81)**

|  |  | **Profession of staff** | | | | | |  |  |
| --- | --- | --- | --- | --- | --- | --- | --- | --- | --- |
| **Training** | **Topic** | **Doctor** | **%** | **Nurse** | **%** | **Total** | **%** | **Likelihood ratio** | ***p*-value** |
|  | **Awareness about Basal-Bolus method** | | | | | | |  |  |
| Trained | Aware | 11 | 42.3 | 15 | 57.7 | 26 | 72.2 | 0.471 | 0.493 |
|  | Unaware | 3 | 30 | 7 | 70 | 10 | 27.8 |  |  |
|  | Total | 14 | 38.9 | 22 | 61.1 | 36 | 44.4 |  |  |
| Untrained | Aware | 4 | 14.3 | 24 | 85.7 | 28 | 62.2 | 0.09 | 0.764 |
|  | Unaware | 3 | 17.6 | 14 | 82.4 | 17 | 37.8 |  |  |
|  | Total | 7 | 15.6 | 38 | 84.4 | 45 | 55.6 |  |  |
| Total n (%) | Aware | 15 | 27.8 | 39 | 72.2 | 54 | 66.7 | 0.289* | 0.591 |
|  | Unaware | 6 | 22.2 | 21 | 77.8 | 27 | 33.3 |  |  |
|  | **Total** | **21** | **25.9** | **60** | **74.1** | **81** | **100** |  |  |
|  | **Awareness about Insulin Infusion method** | | | | | | |  |  |
| Trained | Aware | 4 | 50 | 4 | 50 | 8 | 22.2 | 0.525 | 0.469 |
|  | Unaware | 10 | 35.7 | 18 | 64.3 | 28 | 77.8 |  |  |
|  | Total | 14 | 38.9 | 22 | 61.1 | 36 | 44.4 |  |  |
| Untrained | Aware | 1 | 16.7 | 5 | 83.3 | 6 | 13.3 | 0.006 | 0.936 |
|  | Unaware | 6 | 15.4 | 33 | 84.6 | 39 | 86.7 |  |  |
|  | Total | 7 | 15.6 | 38 | 84.4 | 45 | 55.6 |  |  |
| Total n (%) | Aware | 5 | 35.7 | 9 | 64.3 | 14 | 17.3 | 0.801 | 0.371 |
|  | Unaware | 16 | 23.9 | 51 | 76.1 | 67 | 82.7 |  |  |
|  | **Total** | **21** | **25.9** | **60** | **74.1** | **81** | **100** |  |  |
| **Reasons for lack of awareness about Basal-Bolus method** | | | | | | | | | |
| Medical training (lack of knowledge) | | 5 | 19.2 | 21 | 80.8 | 26 | 96.3 | 3.147 | 0.076 |
| Information overload ( unable to keep up with the guidelines updates) | | 1 | 100 | 0 | 0 | 1 | 3.7 |  |  |
| **Total n (%)** |  | **6** | **22.2** | **21** | **77.8** | **27** | **100** |  |  |
| **Reasons for lack of awareness about Insulin Infusion method** | | | | | | | | | |
| Risk of formal complaint (afraid of responsibility) | | 1 | 100 | 0 | 0 | 1 | 1.5 | 5.929 | 0.52 |
| Not standards of practice ( unusual routine) | | 1 | 100 | 0 | 0 | 1 | 1.5 |  |  |
| Medical training (lack of knowledge) | | 14 | 21.5 | 51 | 78.5 | 65 | 97 |  |  |
| **Total n (%)** |  | **16** | **23.9** | **51** | **76.1** | **67** | **100** |  |  |

*Chi-square test
