# Supplementary material for: Practice of hyperglycaemia control in intensive care units of the Military Hospital, Sudan—Needs of a protocol
Source: PLoS One. 2022 May 24;17(5):e0267655. doi: 10.1371/journal.pone.0267655 (PMC9129021; doi:10.1371/journal.pone.0267655)
Supplement: S2 Table — (DOCX) [file pone.0267655.s002.docx]

**Table S2: Management of diabetic ketoacidosis (DKA) by the participants according to their status of training on glycaemia control (n=81)**

| **DKA Management** | **Doctor** | **%** | **Nurse** | **%** | | **Health Staff** | **%** | **Likelihood ratio** | ***P-value*** |
| --- | --- | --- | --- | --- | --- | --- | --- | --- | --- |
| *Trained* |  |  |  |  |  | |  |  |  |
| Stop the I.V insulin then start the S.C insulin | 4 | 21.1 | 15 | 78.9 | 19 | | 52.8 | 6.627 | 0.036 |
| Overlap the I.V and S.C | 9 | 56.3 | 7 | 43.8 | 16 | | 44.4 |  |  |
| Do not know | 1 | 100 | 0 | 0 | 1 | | 2.8 |  |  |
| ***Total*** | ***14*** | ***38.9*** | ***22*** | ***61*** | ***36*** | | ***100*** |  |  |
| *Untrained* |  |  |  |  |  | |  |  |  |
| Stop the I.V insulin then start the S.C insulin | 4 | 14.8 | 23 | 85.2 | 27 | | 60 | 5.663 | 0.059 |
| Overlap the I.V and S.C | 3 | 37.5 | 5 | 62.5 | 8 | | 17.8 |  |  |
| Do not know | 0 | 0 | 10 | 100 | 10 | | 22.2 |  |  |
| ***Total*** | ***7*** | ***15.6*** | ***38*** | ***84*** | ***45*** | | ***100*** |  |  |
| *Trained and untrained* |  |  |  |  |  | |  |  |  |
| Stop the I.V insulin then start the S.C insulin | 8 | 17.4 | 38 | 82.6 | 46 | | 56.8 | 10.229 | 0.006 |
| Overlap the I.V and S.C | 12 | 50 | 12 | 50 | 24 | | 29.6 |  |  |
| Do not know | 1 | 9.1 | 10 | 90.9 | 11 | | 13.6 |  |  |
| **Total** | **21** | **25.9** | **60** | **74.1** | | **81** | **100** |  |  |
