# Supplementary material for: Practice of hyperglycaemia control in intensive care units of the Military Hospital, Sudan—Needs of a protocol
Source: PLoS One. 2022 May 24;17(5):e0267655. doi: 10.1371/journal.pone.0267655 (PMC9129021; doi:10.1371/journal.pone.0267655)
Supplement: S3 Table — (DOCX) [file pone.0267655.s003.docx]

**Table S3: Practice of measurement of blood glucose by health professional according to their training status (n=81).**

| **Profession** | **BG measurement** | **Training** | | **Total**  **n (%)** | **Chi^2^** | ***p*- value** |
| --- | --- | --- | --- | --- | --- | --- |
|  |  | Yes  n (%) | No  n (%) |  |  |  |
| Doctor | < 6 hourly | 8 (57.1%) | 2 (28.6%) | 10 (47.6%) | 1.567* | 0.211 |
|  | ≥6 hourly | 6 (42.9%) | 5 (71.4%) | 11 (52.4%) |  |  |
|  | Total | 14 (100%) | 7 (100%) | 21 (100%) |  |  |
| Nurse | <6 hourly | 9 (40.9%) | 12 (31.6%) | 21 (35%) | 0.533 | 0.465 |
|  | ≥ 6 hourly | 13 (59.1%) | 26 (68.4) | 39 (65%) |  |  |
|  | Total | 22 (100%) | 38 (100%) | 60 (100%) |  |  |
| Total staff | <6 hourly | 17 (47.2%) | 14 (31.1%) | 31 (38.3%) | 2.197 | 0.138 |
|  | ≥ 6 hourly | 19 (52.8%) | 31 (68.9%) | 50 (61.7%) |  |  |
|  | Total | 36 (100%) | 45 (100%) | 81 (100%) |  |  |

*Likelihood ratio
