# Supplementary material for: Practice of hyperglycaemia control in intensive care units of the Military Hospital, Sudan—Needs of a protocol
Source: PLoS One. 2022 May 24;17(5):e0267655. doi: 10.1371/journal.pone.0267655 (PMC9129021; doi:10.1371/journal.pone.0267655)
Supplement: S4 Table — (DOCX) [file pone.0267655.s004.docx]

**Table S4: Glycaemia measurement methods used the staff of the intensive care units**

|  | **Type of intensive care unit** | | | | | | | |  |  | |  | |  |
| --- | --- | --- | --- | --- | --- | --- | --- | --- | --- | --- | --- | --- | --- | --- |
| **Variable** | **Surgical** | | | **%** | **Mixed** | **%** | **Cardiac** | **%** | **Total staff** | **%** | **Likelihood ratio** | | ***p-value*** | |
| **HbA1c measurement** | | | | | | | | | | | | | | |
| Measured | | | 3 | 5.3 | 46 | 80.7 | 8 | 14.0 | 57 | 69.5 | 12.584 | | 0.002 | |
| Not measured | | | 7 | 28.0 | 18 | 72.0 | 0 | 0.0 | 25 | 30.5 |  | |  | |
| **Total staff** | | | **10** | **12.2** | **64** | **78.0** | **8** | **9.8** | **82** | **100.0** |  | |  | |
| **Hyperglycemia control method** | | | | | | | | | | | | | | |
| Sliding scale | | 9 | | 13.8 | 54 | 83.1 | 2 | 3.1 | 65 | 79.3 |  | |  | |
| Others | | 1 | | 5.9 | 10 | 58.8 | 6 | 35.3 | 17 | 20.7 | 12.728 | | 0.002 | |
| **Total staff** | | **10** | | **12.2** | **64** | **78.0** | **8** | **9.8** | **82** | **100.0** |  | |  | |
