# Supplementary material for: Practice of hyperglycaemia control in intensive care units of the Military Hospital, Sudan—Needs of a protocol
Source: PLoS One. 2022 May 24;17(5):e0267655. doi: 10.1371/journal.pone.0267655 (PMC9129021; doi:10.1371/journal.pone.0267655)
Supplement: S5 Table — (DOCX) [file pone.0267655.s005.docx]

**Table S5: Glycaemia control methods used by healthcare professionals (n=81).**

| **Variable** | **Doctors** | **%** | **Nurses** | **%** | **Total** | **%** | ***p-value**** |
| --- | --- | --- | --- | --- | --- | --- | --- |
| **Hyperglycemia control method** |  |  |  |  |  |  |  |
| Sliding scale method | 15 | 23.4 | 49 | 76.6 | 64 | 79.0 |  |
| Other methods | 6 | 35.3 | 11 | 64.7 | 17 | 21.0 | 0.333 |
| ***Total staff n (%)*** | ***21*** | ***25.9*** | ***60*** | ***74.1*** | ***81*** | ***100.0*** |  |
| **other hyperglycemia control method** | |  |  |  |  |  |  |
| *Basal-Bolus method* | 4 | 28.6 | 10 | 71.4 | 14 | 82.4 |  |
| *Mixed insulin method* | 1 | 50.0 | 1 | 50.0 | 2 | 11.8 | 0.279 |
| *Insulin infusion method* | 1 | 100.0 | 0 | 0.0 | 1 | 5.8 |  |
| ***Total n*** | ***6*** |  | ***11*** |  | ***17*** | 100.0 |  |

*Likelihood ratio.
