# Supplementary material for: Practice of hyperglycaemia control in intensive care units of the Military Hospital, Sudan—Needs of a protocol
Source: PLoS One. 2022 May 24;17(5):e0267655. doi: 10.1371/journal.pone.0267655 (PMC9129021; doi:10.1371/journal.pone.0267655)
Supplement: S6 Table — (DOCX) [file pone.0267655.s006.docx]

**Table S6: Reasons reported by ICU staff and their satisfaction for using a given glycaemia control method**

|  | | **Glycaemia control method** | | | |  |  |  |
| --- | --- | --- | --- | --- | --- | --- | --- | --- |
|  | | **Sliding Scale** | **%** | **Other methods** | **%** | **Total** | **%** | ***p-value*** |
| **Reasons** |  | |  |  |  |  |  |  |
| Standards of practice ( usual routine) | 64 | | 97.0 | 2 | 3.0 | 66 | 80.5 |  |
| Opinion leaders ( the boss said so) | 0 | | 0.0 | 15 | 100.0 | 15 | 18.3 |  |
| Information overload (unable to keep up with the guidelines updates) | 1 | | 100.0 | 0 | 0.0 | 1 | 1.2 | 0.000* |
| **Total** | **65** | | **79.3** | **17** | **20.7** | **82** | **100.0** |  |
| **Staff satisfaction** |  | |  |  |  |  |  |  |
| Yes | 26 | | 76.5 | 8 | 23.5 | 34 | 41.5 |  |
| No | 35 | | 79.5 | 9 | 20.5 | 44 | 53.7 | 0.365** |
| Do not know | 4 | | 100.0 | 0 | 0.0 | 4 | 4.9 |  |
| **Total** | **65** | | **79.3** | **17** | **20.7** | **82** | **100.0** |  |

Likelihood ratio: *65.778, **2.018
