# Supplementary material for: Practice of hyperglycaemia control in intensive care units of the Military Hospital, Sudan—Needs of a protocol
Source: PLoS One. 2022 May 24;17(5):e0267655. doi: 10.1371/journal.pone.0267655 (PMC9129021; doi:10.1371/journal.pone.0267655)
Supplement: S7 Table — (DOCX) [file pone.0267655.s007.docx]

**Table S7: Number of infusion pumps available for each patient across different ICUs**

| **ICU Type** | **ICU name** | **Mean** | **SD** | **Min.** | **Max.** | **F** | ***p*-value** |
| --- | --- | --- | --- | --- | --- | --- | --- |
| **Mixed** | CCR1 | 3.15 | 1.027 | 1 | 6 | 4.953 | 0.001 |
|  | Room-B | 2.86 | 0.864 | 2 | 5 |  |  |
|  | CCR2 | 3.57 | 1.619 | 1 | 6 |  |  |
| **Cardiac** | CCU | 2.25 | 1.389 | 1 | 5 |  |  |
| **Surgical** | Surgery | 1.7 | 0.823 | 0 | 3 |  |  |
