# Supplementary material for: Practice of hyperglycaemia control in intensive care units of the Military Hospital, Sudan—Needs of a protocol
Source: PLoS One. 2022 May 24;17(5):e0267655. doi: 10.1371/journal.pone.0267655 (PMC9129021; doi:10.1371/journal.pone.0267655)
Supplement: S8 Table — (DOCX) [file pone.0267655.s008.docx]

**Table S8: Glycaemic ranges and the hyperglycaemia methods used**

|  | **Glycemic levels** | | | | | | | |  |
| --- | --- | --- | --- | --- | --- | --- | --- | --- | --- |
|  | **Hyperglycaemia (BG>180mg/dl)** | | **Normoglycaemia (BG 71-180 mg/dl)** | | **Hypoglycaemia (BG<71mg/dl)** | | **Total patients** | |  |
|  | **n** | **%** | **n** | **%** | **n** | **%** | **n** | **%** |  |
|  | 10 | 18.5 | 43 | 79.6 | 1 | 1.9 | 54 | 100 |  |
|  | **NICE-SUGAR blood glucose levels** | | | | | | | |  |
|  | **Above range (BG>180mg/dl)** | | **In range (BG 140-180mg/dl)** | | **Below range (BG<140mg/dl)** | | **Total patients** | |  |
|  | **n** | **%** | **n** | **%** | **n** | **%** | **n** | **%** |  |
|  | 10 | 49.1 | 11 | 20.4 | 33 | 61.1 | 54 | 100.0 |  |
|  |  |  |  |  |  |  |  |  |  |
| **Hyperglycemia control method** |  |  |  |  |  |  |  |  | ***p*- value*** |
| Basal- Bolus | 1 | 50.0 | 0 | 0.0 | 1 | 50.0 | 2 | 3.7 | 0.011 |
| Insulin infusion | 0 | 0.0 | 1 | 100.0 | 0 | 0.0 | 1 | 1.9 |  |
| Mixed insulin | 2 | 100.0 | 0 | 0.0 | 0 | 0.0 | 2 | 3.7 |  |
| None | 2 | 5.4 | 9 | 24.3 | 26 | 70.3 | 37 | 68.5 |  |
| Glimepiride | 1 | 100.0 | 0 | 0.0 | 0 | 0.0 | 1 | 1.9 |  |
| Sliding scale | 4 | 36.4 | 1 | 9.1 | 6 | 54.5 | 11 | 20.4 |  |
| **Total patients** | **10** | **18.5** | **11** | **20.4** | **33** | **61.1** | **54** | **100.0** |  |

*Likelihood ratio=22.836
