# Supplementary material for: Practice of hyperglycaemia control in intensive care units of the Military Hospital, Sudan—Needs of a protocol
Source: PLoS One. 2022 May 24;17(5):e0267655. doi: 10.1371/journal.pone.0267655 (PMC9129021; doi:10.1371/journal.pone.0267655)
Supplement: S1 File — (DOCX) [file pone.0267655.s010.docx]

**Military Hospital**

**Pharmaceutical services**

**Intensive care units**

**Local protocol for Hyperglycaemia Control in Intensive Care Units**

**Developed by:**

**Dr. Ghada Omer Hamad Abd El-Raheem - American Board-certified Critical Care Pharmacist**

**Contacts: +24992623636- +249922572830, E-mail:** [**ghadaomer90@gmail.com**](mailto:ghadaomer90@gmail.com)

**Approved by**

**Dr. Kamal Osman Mergani**, Training supervisor in critical care- Intensivist

**Dr. Mudawi Mohammed Ahmed Abdallah**, Medical Director of intensive care units – Clinical Pharmacist

**Dr. Azmi Elshaikh Abdalgani**, General Manager of intensive care units- Intensivist

**Dr. Sami Ahmed Babiker**, Intensivist

**Dr. Selma Ibrahim Elamin**, Head of the department of pharmaceutical services- Clinical Pharmacist

**Endorsed for application:**

**Dr. Selma Ibrahim Elamin**, Head of the department of pharmaceutical services- Clinical Pharmacist

**TABLE OF CONTENTS**

[**1.** **INTRODUCTION** 3](#_Toc96633080)

[**2.** **GLYCEMIC CONTROL IN HYPERGLYCEMIC ADULT CRITICAL CARE PATIENTS (NON-DIABETIC)** 4](#_Toc96633081)

[**3.** **GLYCEMIC CONTROL IN DIABETIC ADULT CRITICAL CARE PATIENTS (TYPE 1, TYPE 2) AND PATIENTS WITH TOTAL PANCREATIC RESECTION** 5](#_Toc96633082)

[**4.** **SWITCHING FROM INTRAVENOUS INSULIN TO ORAL AGENTS OR SUBCUTANEOUS INSULIN** 6](#_Toc96633083)

[**5.** **GLYCEMIC CONTROL IN ACUTE CORONARY SYNDROMES- ACS (STEMI, NSTEMI, UNSTABLE ANGINA)** 8](#_Toc96633084)

[**6.** **DIABETIC KETO- ACIDOSIS (DKA) MANAGEMENT** 10](#_Toc96633085)

[**7.** **HYPEROSMOLAR HYPERGLYCEMIC STATE (HHS) MANAGEMENT** 12](#_Toc96633086)

[**8.** **SPECIAL PATIENTS POPULATION** 13](#_Toc96633087)

[**8.1.** **Patients with renal impairment:** 13](#_Toc96633088)

[**8.2.** **Patients on renal replacement therapy:** 13](#_Toc96633089)

[**8.3.** **Patients on steroid therapy:** 13](#_Toc96633090)

[**8.4.** **Patients on vasopressors:** 13](#_Toc96633091)

[**8.5.** **Patients on total parenteral nutrition (TPN):** 13](#_Toc96633092)

[**8.6.** **Patients undergoing cardiac surgery (CABG):** 14](#_Toc96633093)

[**9.** **RECOMMENDATIONS FOR NURSES** 15](#_Toc96633094)

[**10.** **RECOMMENDATIONS FOR DOCTORS** 16](#_Toc96633095)

[**11.** **FREQUENTLY ASKED QUESTIONS (FAQ)** 17](#_Toc96633096)

[**REFERENCES** 18](#_Toc96633097)

**Hyperglycemia control policy for critically ill patients in intensive care units**

1. **INTRODUCTION**

- In hyperglycemic critically ill or hemodynamically unstable patients, treatment using intravenous insulin infusions is favorable as it enables frequent rate adjustments according to patients’ blood glucose (BG) levels [1]
- For all adult critical care patients in medical Intensive Care Units (ICUs), the target random blood glucose (RBG) is 140- 180 mg/dl [2].
- For patients with ischemic stroke, intra-parenchymal hemorrhage, subarachnoid hemorrhage (SAH), and traumatic brain injury (TBI), the target RBG is 150 mg/dl and absolutely less than 180 mg/dl and avoid RBG less than 100 mg/dl [3, 4].
- Nottingham University Hospitals (NUH) guidelines [5] use the NICE-SUGAR target.
- The systematic review conducted by the American College of Physicians (ACP) concluded that there was no reduction in mortality using intensive insulin therapy for patients with myocardial infarction, acute brain injury, or stroke [6].
- Before starting insulin infusion, you must always check random blood glucose (RBG) twice using different techniques (finger-prick and venous samples). If the RBG level is higher than 180 mg/dl for 2 readings 1 hour apart then insulin infusions might be prescribed [5].
- Insulin infusions are prepared using 50 units of regular insulin (Actrapid); drawn up by insulin syringe; plus 49.5 milliliters of normal saline to make a total volume of 50 milliliters [5].
- Insulin infusions must be accompanied by dextrose-containing fluids or feeding (enteral, parenteral). If fluids and feeding are stopped, insulin infusions must be stopped [5].
- Check RBG when there are new symptoms like sweating, tachycardia, hypotension, or loss of consciousness [5].
- Stable patients who are fully conscious can restart their previous anti-diabetics (subcutaneous insulin or even oral anti-diabetics) 1 hour before stopping insulin infusion. The random blood glucose (RBG) should be measured every 2 hours for the next 6 hours then less frequently [5].
- Do not take the blood samples from the same line that has been receiving glucose-containing fluid [5].
- When the RBG readings are within the target range for 3 consecutive readings (Yale protocol), you can then check it every 6 hours [7].

1. **GLYCEMIC CONTROL IN HYPERGLYCEMIC ADULT CRITICAL CARE PATIENTS (NON-DIABETIC)**

**Nottingham University Hospitals (NUH) [5]**

| **Random blood glucose level ( RBG)** | **Insulin infusion rate** | **Next RBG** |
| --- | --- | --- |
| 180- 216 mg/dl | 1 unit/hour | After 1 hour |
| 217- 270 mg/ dl | 2 units/hour | After 1 hour |
| 271- 324 mg/dl | 3 units/hour | After 1 hour |
| > 324 mg/dl | 4 units/hour and inform medicals | After 1 hour |

**Notes:**

- Higher insulin doses should be given if the RBG readings are always higher than 252 mg/dl.

**Hypoglycemia (Yale protocol)** [7]**:**

| **Random blood glucose level ( RBG)** | **Insulin infusion rate** | **Next RBG reading** |
| --- | --- | --- |
| <50 mg/dl | Discontinue insulin infusion and give 50 ml Dextrose 50% (D50%) IV | Every 15 minutes till RBG>90 mg/dl, then wait 30 minutes and restart insulin infusion at half the rate. |
| 50- 74 mg/dl | Discontinue insulin infusion and give 25 ml of D 50% IV | Every 15 minutes till RBG>90 mg/dl, then wait 30 minutes and restart insulin infusion at half the rate. |
| 75- 99 mg/dl | Discontinue insulin infusion | Every 15 minutes till RBG>90 mg/dl, then wait 30 minutes and restart insulin infusion at 75% of the rate. |

# **GLYCEMIC CONTROL IN DIABETIC ADULT CRITICAL CARE PATIENTS (TYPE 1, TYPE 2) AND PATIENTS WITH TOTAL PANCREATIC RESECTION**

**Nottingham University Hospitals (NUH) [5]**

| **Random blood glucose level ( RBG)** | **Insulin infusion rate** | **Next RBG reading** |
| --- | --- | --- |
| 72- 124.2 mg/dl | 1 unit/hour | After 1 hour |
| 126- 178.2 mg/dl | 2 units/hour | After 1 hour |
| 180- 268.2 mg/ dl | 3 units/hour | After 1 hour |
| 270- 358.2 mg/dl | 4 units/hour | After 1 hour |
| > 360 mg/dl | 5 units/ hour and inform medicals | After 1 hour |

**Notes:**

- Higher insulin doses should be given if the RBG readings are always higher than 252 mg/dl and for patients who were taking high insulin doses.

**Hypoglycemia (Yale protocol)** [7]**:**

| **Random blood glucose level ( RBG)** | **Insulin infusion rate** | **Next RBG** |
| --- | --- | --- |
| <50 mg/dl | Discontinue insulin infusion and give 50 ml Dextrose 50% (D50%) IV | Every 15 minutes till RBG>90 mg/dl, then wait 30 minutes and restart insulin infusion at half the rate. |
| 50- 74 mg/dl | Discontinue insulin infusion and give 25 ml of D 50% IV | Every 15 minutes till RBG>90 mg/dl, then wait 30 minutes and restart insulin infusion at half the rate. |
| 75- 99 mg/dl | Discontinue insulin infusion | Every 15 minutes till RBG>90 mg/dl, then wait 30 minutes and restart insulin infusion at 75% of the rate. |

# **SWITCHING FROM INTRAVENOUS INSULIN TO ORAL AGENTS OR SUBCUTANEOUS INSULIN**

Stable adult diabetic patients can restart their anti-diabetic medications (oral tablets or S.C. insulin) 1 hour before stopping the insulin infusion. The patient must be fed.

Check RBG every 2 hours for at least 6 hours after the first tablet. If the patient restarted the S.C insulin, then RBG should be checked every hour for 2 hours, then 2 hourly for 24 hours after S.C. insulin [5].

Stable non- insulin-requiring patients who were on a 2 units/hour regimen can be discharged without insulin, RBG should be checked within 4 hours after stopping insulin [5].

American college of critical care medicine guidelines recommends that stable critical care patients should be transitioned to a subcutaneous basal-bolus insulin regimen [4]. Switching from intravenous (I.V.) to subcutaneous insulin should be done properly to avoid loss of glycemic control by starting the basal S.C. insulin 1 hour before stopping the insulin infusion.

**Basal- Bolus insulin regimen (subcutaneous regimen):**

- 50%- 60% of the total daily I.V. insulin is the recommended dose of the basal insulin (insulin glargine [Lantus]). In other words, half the I.V. insulin dose in the past 24 hours is the dose of Lantus insulin to be started 1 hour before stopping the insulin infusion, ideally following a meal [8,9]. This method provides good glycemic control with a low risk of hypoglycemia.
- RBG should be measured every 1 hour for 2 hours then every 2 hours for 24 hours.
- For stable patients in which basal-bolus is to be started without knowing the total daily insulin (TDI). We use a weight-based estimate of TDI as follows [10]:

| **Type 1 diabetes** | dose |
| --- | --- |
| Initial dose | 0.3-0.5 unit/Kg/day |
| Honeymoon phase | 0.2-0.5 unit/Kg/day |
| **Type 2 diabetes** |  |
| With insulin resistance | 0.7-1.5 unit/Kg/day |

- Basal insulin: insulin glargine (Lantus). Given once daily, it controls fasting blood glucose. Lantus dose is half the total insulin daily dose.
- Bolus insulin: regular insulin (Actrapid). Given three times daily before meals, it controls post-prandial blood glucose. Ideally, the estimation of bolus insulin doses is done using the “500 rule”, dividing 500 by the total daily insulin (TDI) will estimate the carbohydrate amount in gm that 1 unit of bolus insulin will cover. Otherwise, fixed-dose method can be applied using half the total daily insulin dose divided into 3 doses, and each dose is given before meals [11].

**Correctional insulin needs:**

If fixed-dose did not provide optimal glycemic control and the RBG is still high, then additional doses of regular insulin are needed. The estimated dose to be given is calculated using the “1500 rule” in which [1500/TDI= the number of mg/dl of RBG that are lowered per 1 unit of regular insulin].

For example: if the TDI is 50 units/ day, 1500/50= 30, suggesting that I unit of regular insulin will reduce RBG level by 30 mg/dl.

The advantage of this method is that it mimics natural insulin secretion patterns. Besides, it is more patient-specific than the traditional sliding-scale regimen. Also using Lantus insulin (peak-less long-acting insulin) is less hypoglycemic than using NPH insulin twice daily and more flexible [11].

1. **GLYCEMIC CONTROL IN ACUTE CORONARY SYNDROMES- ACS (STEMI, NSTEMI, UNSTABLE ANGINA)**

**Nottingham University Hospitals (NUH) [12]**

All ACS patients should have their RBG checked on admission. Target RBG is between 72 mg/dl to 198 mg/dl.

The aim is to achieve target RBG either by using intravenous or subcutaneous insulin administered within 4 hours of hospital admission.

**Notes:**

- Hyperglycemia after ACS suggests a high risk of type 2 diabetes.
- Fasting blood glucose should be assessed 4 days after ACS and HbA1c should be checked before discharge.
- If the patient is stable and can take orally (eats food and drinks) then resume home antidiabetics.

**Basal- Bolus insulin regimen (subcutaneous regimen):**

- 50%- 60% of the total daily I.V. insulin is the recommended dose of the basal insulin (insulin glargine{Lantus}). In other words, half the I.V. insulin dose in the past 24 hours is the dose of Lantus insulin to be started 1 hour before stopping the insulin infusion ideally following a meal [8,9]. This method provides good glycemic control with a low risk of hypoglycemia.
- RBG should be measured every 1 hour for 2 hours then every 2 hours for 24 hours.
- For stable patients in which basal-bolus is to be started without knowing the total daily insulin. We use a weight-based estimate of TDI as follows [10]:

| **Type 1 diabetes** | **dose** |
| --- | --- |
| Initial dose | 0.3-0.5 unit/Kg/day |
| Honeymoon phase | 0.2-0.5 unit/Kg/day |
| **Type 2 diabetes** | **dose** |
| With insulin resistance | 0.7-1.5 unit/Kg/day |

- Basal insulin: insulin glargine (Lantus). Given once daily, it controls fasting blood glucose. Lantus dose is half the total insulin daily dose.
- Bolus insulin: regular insulin (Actrapid). Given three times daily before meals, it controls post-prandial blood glucose. Ideally, the estimation of bolus insulin doses is done using the “500 rule”, dividing 500 by the total daily insulin (TDI) will estimate the carbohydrate amount in gm that 1 unit of bolus insulin will cover. Otherwise, the fixed-dose method can be applied using half the total daily insulin dose divided into 3 doses, and each dose is given before meals.

**Correctional insulin needs:**

If fixed-dose did not provide optimal glycemic control and the RBG is still high, then additional doses of regular insulin are needed. The estimated dose to be given is calculated using the “1500 rule” in which [1500/TDI= the number of mg/dl of RBG that are lowered per 1 unit of regular insulin].

For example: if the TDI is 50 units/ day, 1500/50= 30, suggesting that I unit of regular insulin will reduce RBG level by 30 mg/dl.

The advantage of this method is that it mimics natural insulin secretion patterns, hence called insulin therapy. Besides, it is more patient-specific than the traditional sliding-scale regimen. Also using Lantus insulin (peak-less long-acting insulin) is less hypoglycemic than using NPH insulin twice daily and more flexible [11].

1. **DIABETIC KETO- ACIDOSIS (DKA) MANAGEMENT**

**American College of Clinical Pharmacy (ACCP) [3], Applied Therapeutics [10], injectable drugs guide [13]**

**Fluids administration:**

- In the absence of cardiac problems, administer normal saline (NS) with a rate of 1- 1.5 liter during the first hour, then at a rate of 250- 500 ml/hr to target hemodynamic parameters and urine output.
- When random blood glucose (RBG) reaches 200 mg/dl switch fluids to Dextrose ½saline, and if unavailable continue NS infusion and add dextrose 10% infusion at a rate of 125 ml/hour.

**Insulin:**

- Bolus dose: 0.1 units/Kg intravenously I.V.
- Maintenance dose: 0.1/kg/hour I.V.
- If RBG does not decrease by 50 mg/dl in the first hour, double the rate of insulin infusion.
- Measure RBG hourly and titrate insulin infusion to target decrease of RBG by 50- 75 mg/dl/hour.
- Reduce the rate to (0.02-0.05 u/Kg/h) or 0.1 u/Kg/h (if started with double the rate) when RBG reaches 200 mg/dl and switch fluids from NS to dextrose in ½ saline to maintain RBG between 150 – 200 mg/dl **[do not stop the insulin infusion].**
- Once the patient is out of the DKA, give a subcutaneous (S.C.) insulin dose (0.5- 0.8 u/Kg/d) **1 hour before stopping the insulin infusion.**
- Relief of DKA includes the following three criteria: RBG < 200 mg/dl, serum bicarbonate ≥ 15mg/dl and PH > 7.3 [3].

**Potassium (K):**

- If K<3.3 mEq/L, give KCl with a rate of 20- 40 mEq/hour before starting insulin infusion.
- If K> 5.2 mEq/L do not give KCl and check serum potassium every 2 hours, if it falls below 4 mE/L start Kcl infusion.
- If K> 3.3 and < 5.3 mEq/L, give 20- 30 mEq in each liter. In other words, give half an ampoule of KCl with each bottle of fluid to maintain potassium level between 4- 5 mEq/L.

**Bicarbonate: (Consultant decision only)**

- If pH falls below 6.9 (or bicarbonate <10 mEq/L), give Sodium bicarbonate (NaHCO3), 50 ml of NaHCO3 8.4% (1 ampoule) is given via central line over 1 hour [10]. Or by dissolving 100 ml of NaHCO3 8.4% in 400 ml water for injection and 20 mEq KCl by rate 200ml/hour. Once pH is above 7 stop the Bicarbonate infusion [3].

**Phosphorus:**

- Give phosphorous if serum concentration is < 1mg/dl.

**Monitoring:**

- Check RBG hourly and electrolytes every 2 hours to avoid hypokalemia or hypo/hypernatremia.
- Measure ketones (blood or urine) every 1 hour.
- Measure bicarbonate level and pH (arterial blood gas ABG) every 2 hours [10].

1. **HYPEROSMOLAR HYPERGLYCEMIC STATE (HHS) MANAGEMENT**

**American College of Clinical Pharmacy (ACCP) [3], NUH [5]**

**Fluids administration:**

- Fluids only (NS) at the beginning might correct HHS.
- In the absence of cardiac problems, administer normal saline (NS) with a rate of 1- 1.5 Liter during the first hour, then at a rate of 250- 500 ml/hr.
- When random blood glucose (RBG) reaches 300 mg/dl switch to Dextrose ½saline, and if unavailable continue NS infusion and add dextrose 10% infusion at a rate of 125 ml/hour.

**Insulin:**

- Measure RBG hourly and start insulin infusion to target decrease of RBG by 50- 75 mg/dl/hour (< 90 mg/dl/hour). Start at a rate of 0.05 u/Kg/hour
- Reduce the rate to 0.02 U/Kg/h when RBG reaches 300 mg/dl and switch fluids from NS to dextrose in ½ saline, **[do not stop the insulin infusion]** and maintain RBG between 200- 300 mg/dl till mental status changes resolve.
- Once the patient is stable, give a subcutaneous (S.C.) insulin dose (0.5- 0.8 u/Kg/d) **1 hour before stopping the insulin infusion.**

**Potassium (K):**

- If K<3.3 mEq/L, give KCl with a rate of 20- 40 mEq/hour before starting insulin infusion.
- If K> 5.2 mEq/L do not give KCl and check serum potassium every 2 hours, if it falls below 4 mE/L start Kcl infusion.
- If K> 3.3 and < 5.3 mEq/L, give 20- 30 mEq in each liter. In other words, give half an ampoule of KCl with each bottle of fluid to maintain potassium level between 4- 5 mEq/L.

**Additional managements:**

- Treat underlying causes (infection, cardiac events…)
- Administer thrombo-prophylaxis (enoxaparin 40 mg S.C. once daily) if no contraindications.
- Provide foot care and daily checks.
- After weeks to months, the patients can be transferred to oral anti-diabetics.
- Give phosphorous if serum concentration is < 1mg/dl.

**Monitoring:** Check RBG hourly and electrolytes every 2 hours to avoid hypokalemia or hypo/hypernatremia.

1. **SPECIAL PATIENTS POPULATION**
   1. **Patients with renal impairment:**

Patients with renal impairment are more sensitive to insulin because insulin is removed renally. Moderate renal failure patients (GFR > 22.5 ml/min can remove only 39% of endogenous and exogenous insulin. While in severe renal impairment (GFR < 6 ml/min), only 9% of insulin is removed. So when administering insulin, we should pay attention and start with low doses (0.4 of the normal dose up to 0.1 of the normal doses for fourth stage renal impairment) [10].

- 1. **Patients on renal replacement therapy:**

Reduce the dose by 25- 50% and titrate according to response, can be given any time during dialysis

- 1. **Patients on steroid therapy:**

Higher rates of insulin infusions might be needed, because steroids increase insulin resistance [1], increased insulin requirements are about 20% [5].

- 1. **Patients on vasopressors:**

Higher rates of insulin infusions might be needed because Catecholamines increase insulin resistance [1].

- 1. **Patients on total parenteral nutrition (TPN):**
     1. ***Diabetic patients on TPN:***

Give 1 unit of regular insulin for each 10 gm of dextrose used in the preparation of the TPN mixture. This dose of insulin is either added to the TPN mixture or given in a separate infusion.

If the BG level is still high (above 140 mg/dl), then increase the rate of infusion by 0.5 units for each 10 gm of dextrose [1].

- - 1. ***Non-diabetic hyperglycemic patients on TPN:***

Give 1 unit of regular insulin for each 20 gm of dextrose used in the preparation of the TPN mixture. This dose of insulin is either added to the TPN mixture or given in a separate infusion [1].

If the BG is still high (above 140 mg/dl) increase the rate to be 1 unit per 15 gm of dextrose.

- 1. **Patients undergoing cardiac surgery (CABG):**

Tight glycemic control for 3 postoperative days has been shown to have benefits more than risks [14, 15], but ACP stated that no mortality benefit of tight insulin therapy during the perioperative period [6].

For diabetic patients undergoing cardiac surgery, the GLUCO- CABG trial showed no difference in morbidity or mortality between tight (110-140 mg/dl) and conventional glucose levels (140- 180 mg/dl) [16].

American college of critical care medicine recommends a BG target of less than 150 mg/dl for cardiac surgery patients [4].

In conclusion, the NICE-SUGAR target is optimum for diabetic patients undergoing coronary artery bypass graft surgery [16].

1. **RECOMMENDATIONS FOR NURSES**

- Before starting insulin infusion always check RBG twice using different techniques (finger-prick and venous samples). If RBG level is higher than 180 mg/dl for 2 readings 1 hour apart then insulin infusions may be prescribed [5].
- Insulin infusions are made using 50 units Actrapid insulin (drawn up by insulin syringe) plus 49.5 ml of normal saline to make a total volume of 50 milliliters [5].
- Flush 20 milliliters of the made fluid through the I.V. set first before administration because insulin adheres to the infusion bag and set [10].
- Change the insulin infusion every 24 hours and the infusion set and cannula every 72 hours [13].
- Insulin infusions must be accompanied by dextrose-containing fluids or feeding (enteral, parenteral), if fluids and feeding stopped insulin infusions must be stopped [5].
- Do not take the blood samples from the line that contains glucose-containing fluid.
- When the RBG readings are within the target for 3 consecutive readings (Yale protocol), you can then check it every 6 hours.
- Check RBG when there is new sweating, tachycardia, hypotension, or loss of consciousness.

1. **RECOMMENDATIONS FOR DOCTORS**

- Note that patients on insulin infusions are at risk of developing hypokalemia, so supplementary potassium might be needed for most patients except hyperkalemic patients and patients with eGFR< 15ml/min/1.73 m^2^ [9].

| Criteria | Infusion fluid | Additives | Rate (ml/hr) |
| --- | --- | --- | --- |
| K< 4.9mmol/L  (eGFR>15ml/min/1.73 m^2^ | Dextrose 5%  1000 ml | KCl 40 mmol | 100ml/hr |
| K< 4.9mmol/L  ( on fluid restriction) | Dextrose 10%  500 ml | KCl 20 mmol | 50ml/hr |
| K>5 mmol/L | Dextrose 10%  500 ml | NONE | 50ml/hr |
| eGFR<15ml/min/1.73 m^2^ or dialysis | Dextrose 10%  500 ml | NONE | 50ml/hr |

- Prescribe long-acting insulin [Lantus] along with the insulin infusion. If the admitted patient is taking Lantus, keep administering it at the usual dose and time, and also for all hyperglycemic patients for better control of BG during insulin infusion Lantus is better to be given [9].
- Do not forget that insulin infusions should be stopped 1 hour after starting the subcutaneous insulin [8, 9].
- Do not forget administering dextrose-containing fluids (or substrates) while administering insulin infusions [8,9].

1. **FREQUENTLY ASKED QUESTIONS (FAQ)**
2. **Do we give K supplementation in fluids extra to the maintenance fluids the patient is currently taking?**

No, give K in the maintenance fluids, give 40 mmol (2 ampules of KCl) in 1000 ml of maintenance fluids (40 mmol/day).

1. **Can we give K in dextrose containing fluids?**

Yes.

1. **If the patient’s RBG is high, can we give insulin boluses along with the infusion?**

Yes, but there is a higher risk of hypoglycemia. We can give boluses of I.V regular insulin with the consultation of the doctor and monitoring RBG every 30 minutes. (Portland Protocol) [7]. The following table can be used for administering insulin boluses, but this algorithm provides tighter control (80-120 mg/dl).

| **Blood Glucose (mg/dl)** | **Bolus I.V regular insulin** |
| --- | --- |
| 181-240 | 6 units |
| 241-300 | 8 |
| 301-360 | 12 |
| >360 | 16 |

1. **Can we give S.C. insulin glargine along with the insulin infusion?**

Yes, and it is recommended as it provides better control without the risk of hypoglycemia, if the patient is already taking it continue with the same dose, and if it is to be started calculate the dose estimation using weight [10].

1. **What if all control measures did not get the RBG to the Target levels?**

We can use (WU) algorithms 3 or 4 after **consultation of an endocrinologist**

| **Blood Glucose (mg/dl)** | **Algorithm-3 (Units/hour)** | **Algorithm-4 (Units/hour)** |
| --- | --- | --- |
| 180-209 | 5 | 9 |
| 210-239 | 6 | 12 |
| 240-269 | 8 | 16 |
| 270-299 | 10 | 20 |
| 300-329 | 12 | 24 |
| 330-359 | 14 | 28 |
| >360 | 16 |  |

# **REFERENCES**

1. Gosmanov AR, Umpierrez GE. Management of hyperglycaemia during enteral and parenteral nutrition therapy. Curr Diab Rep.2013; 13(1): 155-162.
2. Finfer S, Chittock D, et al. Intensive versus conventional glucose control in critically ill patients with traumatic brain injury: long-term follow-up of a subgroup of patients from the NICE-SUGAR study. Intensive Care Med. 2015;41(6):1037-1047. doi:10.1007/s00134-015-3757-6
3. Dickerson R. Fluids, electrolytes, acid-base disorders, and nutrition support. In: Bass S, Benken ST, et al., eds. Critical Care Pharmacy Preparatory Review and Recertification Course, 2019 ed. Lenexa, KS: American College of Clinical Pharmacy, 2019: P 83- 137.
4. Bass SN, Bauer SR. Hepatic failure/GI/endocrine emergencies. In: Bass S, Benken ST, et al., eds. Critical Care Pharmacy Preparatory Review and Recertification Course, 2019 ed. Lenexa, KS: American College of Clinical Pharmacy, 2019: P 569- 626.
5. Alrifai Z, Beed M, Javaid A, Jamieson E. Management and monitoring of insulin infusions and glycemic control in adult critical care patients. Nottingham University Hospitals NHS; 3. 2020. P 19.
6. Qaseem A, Humphrey LL, Chou R, Snow V, Shekelle P; Clinical Guidelines Committee of the American College of Physicians. Use of intensive insulin therapy for the management of glycemic control in hospitalized patients: a clinical practice guideline from the American College of Physicians. Ann Intern Med. 2011;154(4):260-267. Doi:10.7326/0003-4819-154-4-201102150-00007
7. Steil, G. M., Deiss, D., Shih, J., Buckingham, B., Weinzimer, S., & Agus, M. S. D. (2009). Intensive Care Unit Insulin Delivery Algorithms: Why So Many? How to Choose? Journal of Diabetes Science and Technology, 3(1), 125–140. <https://doi.org/10.1177/193229680900300114>.
8. Doolin MK, Walroth TA, Harris SA, Whitten JA, Fritschle-Hilliard AC. Transition From Intravenous to Subcutaneous Insulin in Critically Ill Adults. J Diabetes Sci Technol. 2016;10(4):932-938. Published 2016 Jun 28. Doi:10.1177/1932296816629985
9. Clayton J. Guideline for Variable Rate Insulin Infusion (VRIII) prescription and supplementary fluid prescription. Nottingham University Hospitals NHS. 2018; 2: P 12.
10. Alldredge, B. K., Corelli, R. L., Ernst, M. E., Guglielmo, B. J., Jacobson, P. A., Kradjan, W. A., & Williams, B. R. (2013). Koda-Kimble and Young's applied therapeutics: The clinical use of drugs. Wolters Kluwer Health Adis (ESP).
11. Irons BK. Endocrine and metabolic disorders. ACCP updates in therapeutics: The pharmacotherapy preparatory review and recertification course. 2019; volume (1): p 655-708.
12. Erhayiem B. Guidance based on NICE clinical guideline 130 ‘Management of hyperglycaemia in acute coronary syndromes’ - Issued: October 2011 - [www.nice.org.uk/cg130](http://www.nice.org.uk/cg130).
13. Gray A, Write J, Googey V, Bruce L. Injectable drugs guide. Pharmaceutical Press. London. 2011. ISBN 978 0 85369 787 9.
14. Klonoff DC. Intensive insulin therapy in critically ill-hospitalized patients: making it safe and effective. J Diabetes Sci Technol; 2011: 755-767. Doi:10.1177/193229681100500330
15. Furnary AP. Clinical benefits of tight glycaemic control: focus on the perioperative setting. Best Pract Res Clin Anaesthesiol. 2009; 23 (4): 411-420.
16. Umpierrez G, Cardona S, Pasquel F, et al. randomized controlled trial of intensive versus conservative glucose control in patients undergoing coronary artery bypass graft surgery: GLUCO-CONTROL Trial. 2015; 38: 1665-1672.
