# Supplementary material for: Practice of hyperglycaemia control in intensive care units of the Military Hospital, Sudan—Needs of a protocol
Source: PLoS One. 2022 May 24;17(5):e0267655. doi: 10.1371/journal.pone.0267655 (PMC9129021; doi:10.1371/journal.pone.0267655)
Supplement: Attachment — Submitted filename: Response to reviewers March 2022.docx [file pone.0267655.s011.docx]

**Response to Reviewer**

Dear Reviewer,

Thank you for dedicating you valuable time to conduct a critical appraisal for our submitted manuscript. It is our pleasure to submit to PLOS ONE the manuscript titled “Practice of Hyperglycaemia Control in Intensive Care Units of the Military Hospital, Sudan - Needs of a Protocol”. The authors received no funding for this work. We chose PLOS ONE to publish our manuscript, because of the powerful peer review process. We have addressed all the following points:

- Ethical concerns.
- Data availability.
- Organising the results to be more clear.
- Explaining why DKA blood glucose readings were excluded.
- Rewriting the discussion and conclusion sections as well as the study limitations.
- Uploaded the figure to PACE tool and the hyperglycemia protocol to protocols.io

All the supporting data were uploaded as supporting information with no legal or ethical restrictions.

Thank you again for your valuable time and priceless advices.

With our best regards, we remain.

Ghada Omer Hamad Abd El-Raheem
